# Discovery of Novel Symmetrical 1,4-Dihydropyridines as Inhibitors of Multidrug-Resistant Protein (MRP4) Efflux Pump for Anticancer Therapy

**DOI:** 10.3390/molecules26010018

**Published:** 2020-12-22

**Authors:** Henry Döring, David Kreutzer, Christoph Ritter, Andreas Hilgeroth

**Affiliations:** 1 Research Group of Drug Development, Institute of Pharmacy, Martin Luther University Halle-Wittenberg, 06120 Halle, Germany; henry.doering@arcor.de (H.D.); david.kreutzer94@web.de (D.K.); 2 Department of Clinical Pharmacy, Institute of Pharmacy, Ernst Moritz Arndt University Greifswald, 17489 Greifswald, Germany; ritter@uni-greifswald.de

**Keywords:** anticancer drug, drug resistance, structure activity, inhibition, substituent

## Abstract

Despite the development of targeted therapies in cancer, the problem of multidrug resistance (MDR) is still unsolved. Most patients with metastatic cancer die from MDR. Transmembrane efflux pumps as the main cause of MDR have been addressed by developed inhibitors, but early inhibitors of the most prominent and longest known efflux pump P-glycoprotein (P-gp) were disappointing. Those inhibitors have been used without knowledge about the expression of P-gp by the treated tumor. Therefore the use of inhibitors of transmembrane efflux pumps in clinical settings is reconsidered as a promising strategy in the case of the respective efflux pump expression. We discovered novel symmetric inhibitors of the symmetric efflux pump MRP4 encoded by the ABCC4 gene. MRP4 is involved in many kinds of cancer with resistance to anticancer drugs. All compounds showed better activities than the best known MRP4 inhibitor MK571 in an MRP4-overexpressing cell line assay, and the activities could be related to the various substitution patterns of aromatic residues within the symmetric molecular framework. One of the best compounds was demonstrated to overcome the MRP4-mediated resistance in the cell line model to restore the anticancer drug sensitivity as a proof of concept.

## 1. Introduction

Cancer is an ongoing burden for humanity with increasing costs for therapy of patients [1,2,3]. Older chemotherapeutic agents affected all dividing human cells and had strong side effects [4,5]. However, besides tumor surgery as the best alternative, they were the only weapon to combat tumor growth. Meanwhile, specific targeting drugs have been developed which address tumor-specific structures [4]. They cause fewer side effects but have a limited use only for special kinds of cancer in single cases [4]. Most prominent drugs have been monoclonal antibodies, but they are expensive, and their benefit is limited due to a partial short elongation of life for the patients [6,7]. Protein kinase inhibitors are established alternatives with lower costs but increasing resistances as a result of single mutations that reduce the drug-binding affinities [8]. Inhibitors emerged with more than one protein kinase as a target structure [9]. However, these inhibitors are also affected by resistance mechanisms [10].

Besides the various resistance mechanisms that occur toward single drugs, multidrug resistance (MDR) is the main obstacle in cancer therapy [11,12]. Transmembrane efflux pumps are known as causative agents in most cases of MDR [11,13]. They pump anticancer drugs that were taken up by the cancer cells out of the cells so that the cell becomes resistant toward the treatment with the used drug [13]. Moreover, the used drug itself induces the expression of a respective efflux pump and thus the cancer cell resistance [13,14]. Due to a discussed multivalent drug-binding site, several drugs of different structures are affected by the cancer cell resistance and make it a multidrug resistance [15,16]. P-glycoprotein was the earliest discovered efflux pump, followed by the multidrug resistance proteins (MPRs) MRP1 and MRP2 and the breast cancer resistance protein BCRP [15,16]. In order to identify promising drugs that may defeat the activity of the transmembrane efflux pumps, known drugs were screened in efflux pump expressing cells as modulators to overcome the resistance [11,17]. First P-gp inhibitors were identified that were partly toxic and could not be used due to their own pharmacological properties [17]. Second- and third-generation P-gp inhibitors followed with encouraging in vitro results but were disappointing in clinical settings [11,18,19]. Recent studies proved that in those early cases the real expression of P-gp was not investigated and detected. In the case of such expression, studies proved a correlation of the expression rate and anticancer drug resistance and successful treatment with effective P-gp inhibitors [11,20,21]. Moreover, the use of protein kinase inhibitors in co-application with anticancer drugs showed a benefit due to such modulation of a transmembrane efflux pump activity [11,22,23]. Contrasting the known efflux pumps P-gp, MRP1, and MRP2, MRP4 displays a symmetrical molecular framework and recently became an attractive target for cancer therapy because it transports several anticancer drugs not similar to the older known efflux pumps [24,25]. Expression of MRP4 was recently found in osteosarcoma, lymphoma, neuroblastoma, medulloblastoma as well as epithelial ovarian, clear-cell renal cell, and pancreatic cancer, where it has been associated with cell proliferation, tumor growth, and aggressiveness of the disease [26,27,28,29,30,31,32,33]. The regulation of critical pathways that drive the disease is attributed to the unique ability of MRP4 to transport endogenous signaling molecules such as cAMP, which also might affect the transporter’s suggested impact on cellular differentiation processes within the hematopoietic system [34,35,36]. The anticancer drug substrate spectrum of MRP4 focuses on but is not limited to nucleoside analogs such as 6-mercaptopurine, cytarabine, or capecitabine and also includes arsenite, irinotecan, and methotrexate [31,37,38,39,40]. Its role in drug resistance to these anticancer drugs is underscored by the discovery of genetic variants that due to their impaired activity were able to revert the drug resistance phenotype [38,41,42].

We discovered symmetric 1,4-dihydropyridines as novel inhibitors of the symmetric efflux pump MRP4 that plays a role in various types of solid tumors [37]. Early 1,4-dihydropyridines of the nifedipine type with non-substituted nitrogen showed weak inhibitions of P-gp [43]. As they were active as calcium-antagonists with the unsubstituted nitrogen, they could not be used as efflux pump inhibitors. Thus we synthesized *N*-benzyl substituted compounds as potential efflux pump inhibitors with a symmetric molecular scaffold to inhibit the symmetric efflux pump MRP4 encoded by the ABCC4 gene. As only a few inhibitors of MRP4 are known [25,37], we developed our novel 1,4-dihydropyridines with varying substitution patterns to investigate their influence on the transporter activity. The activity was evaluated in a cellular assay of an MRP4-overexpressing cell line by the use of the fluorescent MRP4 substrate calcein as will be discussed. Substituent-dependent effects on the activity were found, and one of the best compounds was evaluated to resensitize the resistant cell line toward the cytostatic drug 6-mercaptopurine as a proof of principle.

## 2. Results and Discussion

### 2.1. Formation of the 1,4-Dihydropyridines

**Scheme 1:**
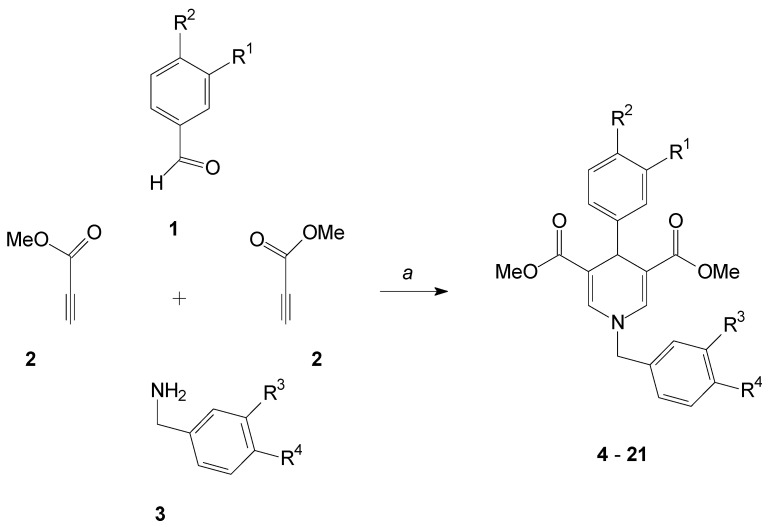
Formation of the 1,4-dihydropyridines **4**–**21**. a: 80 °C, acetic acid.

Known inhibitors of transmembrane efflux pumps as natural or synthetic compounds like cyclosporine or valspodar own complicated molecular structures that are partly difficult to synthesize. Our 1,4-dihydropyridines as novel MRP4 inhibitors are accessible in a simple one-pot reaction of three components, namely an aromatic aldehyde **1**, methyl propiolate **2,** and a benzylamine compound **3** as shown in Scheme 1. Thus the synthesis is different from the known Hantzsch-synthesis of 1,4-dihydropyridines in that a dicarbonyl compound is used instead of methyl propiolate [44]. Our target compounds **4**-**21** own no substituents in the 2- and 6-positions where those formed with the dicarbonyl compound own alkyl substituents at the respective 1,4-dihydropyridine positions.

After finishing the reaction that was followed by thin-layer chromatography (TLC), the solvent was removed. The compound purification and final isolation led to solid products that were spectroscopically characterized to confirm the given structures with proton signals for the hydrogen atoms at the 2- and 6-positions at about 7.45 ppm and for the 4-hydrogen atom at about 4.75 ppm in the ^1^H NMR spectra.

### 2.2. MRP4 Efflux Pump Inhibition with the 1,4-Dihydropyridines

However, due to the recent promotion of MRP4 to an anticancer target, there is a certain lack of MRP4 inhibitors. From those few inhibitors with low potency, the most prominent one is the cysteinyl leukotriene receptor 1 antagonist MK571 that shows additional pharmacological activities as a phosphodiesterase inhibitor [26,45]. Thus, novel inhibitors are necessary.

MRP4 has a symmetric molecular framework built of two transmembrane domains (TMDs) that each consist of six α-helical subunits surrounding the inner channel for outward transport of the MRP4 substrates [46,47]. The substrate-binding cavity for outward transport consists of amino acid residues with arranged subunits similar to the symmetric P-gp [47]. Therefore, we wondered whether our symmetric 1,4-dihydropyridines may serve as promising inhibitors of the MRP4 activity.

In our assay system, we determined the uptake of the fluorescent MRP4 substrate calcein in the pancreatic colo357 and the colo357 MRP4-overexpressing cell lines both with and without our 1,4-dihydropyridines as potential inhibitors. In the case of inhibition of MRP4, an increase of the uptake of the fluorescent MRP4 substrate calcein was observed in the MRP4-overexpressing cell line. The fluorescence amount of the MRP4 substrate was measured for the inhibited cell lines where it increased only in the MRP4-overexpressing cell line due to the inhibition while in the non-inhibited cells fluorescence remained unchanged. A difference in fluorescence was calculated from these values for each cell line. The given values were divided to result in a fluorescence activity ratio (*FAR*). The *FAR* values are shown in Table 1. The higher the inhibition effect was, the higher were the resulting *FAR* values.

We started with a *meta* trifluoromethyl substitution of the 4-phenyl residue and combined it with *N*-benzyl ring substitutions in derivatives **4**–**7**. Both *meta* trifluoromethyl 4-phenyl and *N*-benzyl substituted compound **4** with a *FAR* value of 1.28 was much more active than the used MRP4 inhibitor MK571 for that a value of 0.82 was determined. When the *meta* trifluoromethyl substituent in the *N*-benzyl residue was replaced with a methoxy substituent, the activity of compound **5** was reduced. If that methoxy substituent moved to the *para* position in compound **6**, a further reduction of activity was found. A combination of both a *meta* and *para* methoxy *N*-benzyl substitution in derivative **7** led to an almost unchanged activity.

If the trifluoromethyl substitution in the 4-phenyl residue moved to the *para* position, the activity of the resulting compound **8** was found to increase compared to derivative **5** with both the trifluoromethyl and methoxy substituent in the *meta* position of the aromatic residues. When the *meta* methoxy substitution in the *N*-benzyl substituent moved to the *para* position, the activity of compound **9** decreased. A combination of both *meta* and *para* methoxy *N*-benzyl substitutions in compound **10** resulted in a slightly decreased activity compared to the only *meta* substituted compound **8**. It can be concluded that the *meta* substitution of the *N*-benzyl ring is more favorable than the *para* substitution with all compounds showing a better activity than MK571.

Next, we distributed the fluoro functions from the trifluoro substituent to the *meta* and *para* functions of the 4-phenyl residue in compounds **11**–**13**. Combined with the *meta* methoxy function at the *N*-benzyl phenyl ring in compound **11**, we reached an increase in activity compared to both the trifluoromethyl substituted derivatives **5** and **8**. If the methoxy function moved to the *para* position of the *N*-benzyl residue, a slight decrease resulted for compound **12**. A combination of a *para* und *meta* methoxy substitutions in the *N*-benzyl residue of derivative **13** also resulted in a favorable activity compared to the two trifluoromethyl compounds **7** and **10.** The only *meta* fluoro 4-phenyl substituted derivative **14** and the just *para* fluoro substituted compound **15** were less active than the difluoro 4-phenyl compound **13**. Therefore it can be concluded that the disubstitution of the 4-phenyl ring resulted in the best activities so far.

Next, we combined a *para* methoxy and a *meta* trifluoromethyl residue as disubstitution at the 4-phenyl substituent with the respective *N*-benzyl ring substituents of the first compounds **4**–**7**. The *meta* trifluoromethyl *N*-benzyl substitution in derivative **16** resulted in the almost similar activity of both trifluoromethyl substitutions in compound **4**. If the *meta* trifluoro substitution in the *N*-benzyl ring was replaced with a methoxy substituent, we found the main increases in activity for derivative **17** with a *FAR* value of 1.55. If that *meta* methoxy function moved to the *para* position in compound **18**, we found a decreased activity, and both *meta* and *para* methoxy substituted compound **19** resulted in an activity lower than that of derivative **17** and better than that of compound **18**. Again, the disubstitution of the 4-phenyl ring resulted in improved activities with the *meta* substitution in the *N*-benzyl ring being more favorable than the *para* position.

Finally, we investigated a dimethoxy function in the 4-phenyl ring and combined it with the *meta* trifluoro substituent of the *N*-benzyl phenyl ring in compound **20**. Compared to derivative **16**, the activity was found to decrease. When the dimethoxy substitution was replaced with a dibenzyloxy function, the activity of compound **21** was found to increase.

Generally, the disubstituted 4-phenyl compounds with a *meta* substitution in the *N*-benzyl phenyl ring resulted in the best MRP4 inhibiting activities. In order to gain a first insight into an MRP4-specific inhibition effect, we determined the corresponding *FAR* values for the best MRP4-inhibiting compounds **11**, **12**, **17,** and **21** in an MRP1-overexpressing ovarian carcinoma cell line [48]. Compared to the used MRP1 standard probenecid for which a *FAR* value of 1.23 was determined at a used concentration of 10 µM, we found partly mainly reduced values of 0.92 for compound **11**, 0.97 for compound **12**, 1.01 for compound **17**, and finally 0.82 for compound **20**. If compared to the compounds MRP4 activities in relation to the MK571 standard that means just a low activity toward MRP1.

### 2.3. In Vitro MRP4 Resistance Studies of Drug Reversal

MRP4 has been associated with various kinds of cancer due to an observed overexpression that was mostly based on determined mRNA analysis. Described compound effects on MRP4 efflux inhibition are rare and mostly limited to drugs that were discovered from compound libraries with pharmacological properties different from the MRP4 inhibition, therefore a perspective use for therapy was excluded [23,37]. As recently reported for efflux pump inhibitors for MDR cancer therapy, their use will be effective if the respective efflux pump is expressed by the tumor tissue to be really blocked by the inhibitor so that the anticancer drug resistance mediated by that efflux pump can be overcome.

In order to perspectively profile our novel compound class of effective MRP4 inhibitors, we further evaluated the inhibitor ability to resensitize the colo357 MRP4-overexpressing cell line toward a cytostatic drug as MRP4 substrate. We used 6-mercaptopurine as antimetabolite in cancer therapy and MRP4 substrate [25] and determined the toxicity of 6-mercaptopurine in the colo357 and the MRP4-overexpressing cell line in an MTT assay to detect the cellular toxicity as a result of damage of the mitochondrial activity of MTT reduction.

In the anticancer drug-sensitive cell line colo357, we determined an IC_50_ value of 24.88 µM for 6-mercaptopurine. In the MRP4-overexpressing cell line, the IC_50_ value was determined with 224.0 µM which means a loss of sensitivity for 6-mercaptopurine. Then we determined the toxicity in the MRP4-overexpressing cell line with the use of 10 µM of the MRP4 inhibitor MK571. The IC_50_ value of 6-mercaptopurine was determined with 313.6 µM which practically means no effect of an efflux pump inhibition that would have reduced the IC_50_ values of mercaptopurine. Then we used our best MRP4 inhibitor compound **17** from our uptake assay studies. We determined an IC_50_ value for mercaptopurine of 33.73 µM. That IC_50_ value almost reached the value determined for mercaptopurine in the non-MRP4-expressing colo357 cell line. Cellular toxic effects of compound **17** itself and the other derivatives could be excluded by previous studies of FACS analyses with the used compound preincubated cell lines that would have indicated such cellular effects in the cell sorting process.

Therefore we found that the anticancer drug sensitivity was restored by the use of our best inhibitor as a proof of principle.

## 3. Material and Methods

### 3.1. Chemical Reagents and Instruments

Commercial reagents were used without further purification. The ^1^H-NMR spectra (500 MHz) were measured using tetramethylsilane as an internal standard. Thin-layer chromatography (TLC) was performed on E. Merck 5554 silica gel plates. The high-resolution mass spectra were recorded on a Finnigan LCQ Classic mass spectrometer (Mudelein, IL, USA).

### 3.2. General Procedure for the Synthesis of Compounds **4**–**21**

One equivalent of the aromatic aldehyde **1**, two equivalents of the methyl propiolate **2**, and one equivalent of the benzylamine **3** were dissolved in 2 mL of freshly distilled acetic acid and heated under reflux under stirring at 80 °C. The reaction procedure was followed by TLC and mass spectrometry. After finishing, a brine solution was added, and the mixture was extracted with chloroform several times. The unified organic layer was reduced in volume, and residual acetic acid was removed with toluene. Then methanol or a mixture of diethyl ether and ethanol was added under cooling to give the final product under crystallization. In the case of non-crystallizing, the reaction mixture had to be purified by column chromatography using silica gel and eluent mixtures of cyclohexane and ethyl acetate. The unified compound-containing fractions were evaporated, and again methanol or a mixture of diethyl ether and methanol was added and the purified compound crystallized.

Dimethyl N-(3-trifluoromethylbenzyl)-4-(3-trifluoromethylphenyl)-1,4-dihydropyridine-3,5-carboxylate (**4**). Yield 15%, yellow powder; mp 99–101 °C; ^1^H NMR (DMSO-d_6_) δ = 3.54 (s, 6H, COOCH_3_), 4.80 (s, 1H, 4-H), 4.92 (s, 2H, CH_2_), 7.29 (s, 1H, 2-H of phenyl), 7.37–7.44 (m 2H, 4-H of phenyl and benzyl), 7.46–7.50 (m, 1H, 5-H of phenyl), 7.62 (s, 2H, 2-, 6-H), 7.637.68 (m, 2H, 6-H of phenyl and benzyl), 7.68–7.73 (m, 1H, 5-H of benzyl), 7.76 (s, 1H, 2-H of benzyl); *m*/*z* (ESI) 522.48 (M+Na^+^).

Dimethyl N-(3-methoxybenzyl)-4-(3-trifluoromethylphenyl)-1,4-dihydropyridine-3,5-carboxy-late (**5**). Yield 8%, yellow-red powder; mp 104–105 °C; ^1^H NMR (DMSO-d_6_) δ = 3.53 (s, 6H, COOCH_3_), 3.74 (s, 3H, OCH_3_), 4.77 (s, 2H, CH_2_), 4.79 (s, 1H, 4-H), 6.87–6.95 (m, 3H, 3-, 4-, 6-H of benzyl), 7.31 (t, J = 7.8 Hz, 1H, 5-H of benzyl), 7.35 (s, 1H, 2-H of phenyl), 7.39–7.45 (m, 2H, 5-, 6-Hof phenyl), 7.46–7.50 (m, 1H, 4-H of phenyl), 7.54 (s, 2H, 2-, 6-H); *m*/*z* (ESI) 484.49 (M + Na^+^).

Dimethyl N-(4-methoxybenzyl)-4-(3-trifluoromethylphenyl)-1,4-dihydropyridine-3,5-carboxy-late (**6**). Yield 15%, yellow crystals; mp 101–103 °C; ^1^H NMR (DMSO-d_6_) δ = 3.53 (s, 6H, COOCH_3_), 3.74 (s, 3H, OCH_3_), 4.73 (s, 2H, CH_2_), 4.78 (s, 1H, 4-H), 6.94 (dd, J = 6.6, 2.0 Hz, 2H, 3-, 5-H of benzyl), 7.29 (dd, J = 6.6, 2.1 Hz, 2H, 2-, 6-H of benzyl), 7.32 (s, 1H, 2-H of phenyl), 7.36–7.50 (m, 3H, 4-, 5-, 6-H of phenyl), 7.52 (s, 2H, 2-, 6-H); *m*/*z* (ESI) 484.52 (M + Na^+^).

Dimethyl N-(3,4-dimethoxybenzyl)-4-(3-trifluoromethylphenyl)-1,4-dihydropyridine-3,5-carboxylate (**7**). Yield 24%, yellow solid; mp 131–133 °C; ^1^H NMR (DMSO-d_6_) δ = 3.53 (s, 6H, COOCH_3_), 3.73, 3.74 (2 × s, 2 × 3H, OCH_3_), 4.70 (s, 2H, CH_2_), 4.79 (s, 1H, 4-H), 6.88 (dd, J = 8.2, 2.0 Hz, 1H, 6-H of benzyl), 6.95 (d, J = 8.2 Hz, 1H, 5-H of benzyl), 7.00 (d, J = 2.0 Hz, 1H, 2-H of benzyl), 7.35 (s, 1H, 2-H of phenyl), 7.37-7.44 (m, 2H, 4-, 6-H of phenyl), 7.43–7.51 (m, 1H, 5-H of phenyl), 7.53 (s, 2H, 2-, 6-H; *m*/*z* (ESI) 514.63 (M + Na^+^).

Dimethyl N-(3-methoxybenzyl)-4-(4-trifluoromethylphenyl)-1,4-dihydropyridine-3,5-carboxylate (**8**). Yield 10%, yellow-orange solid; mp 116–117 °C; ^1^H NMR (DMSO-d_6_) δ = 3.53 (s, 6H, COOCH_3_), 3.74 (s, 3H, OCH_3_), 4.77 (s, 2H, CH_2_), 4.79 (s, 1H, 4-H), 6.87–6.94 (m, 3H, 3-, 4-, 6-H of benzyl), 7.31 (d, J = 8.0 Hz, 2H, 2-, 6-H of phenyl), 7.32–7.36 (m, 1H, 5-H of benzyl), 7.52 (s, 2H, 2-, 6-H), 7.54 (d, J = 8.0 Hz, 2H, 3-, 5-H of phenyl); *m*/*z* (ESI) 484.74 (M + Na^+^).

Dimethyl N-(4-methoxybenzyl)-4-(4-trifluoromethylphenyl)-1,4-dihydropyridine-3,5-carboxylate (**9**). Yield 26%, yellow-white solid; mp 129–130 °C; ^1^H NMR (DMSO-d_6_) δ = 3.52 (s, 6H, COOCH_3_), 3.75 (s, 3H, OCH_3_), 4.72 (s, 2H, CH_2_), 4.77 (s, 1H, 4-H), 6.96–6.99 (m, 2H, 3-,5-H of benzyl), 7.25–7.33 (m, 2h, 2-, 6-H of benzyl), 7.29 (d, J = 8.4 Hz, 2H, 2-, 6-H of phenyl), 7.51 (s, 2H, 2-, 6-H), 7.53 (d, J = 8.2 Hz, 2H, 3-, 5-H of phenyl); *m*/*z* (ESI) 461.40 (M^+^).

Dimethyl N-(3,4-dimethoxybenzyl)-4-(4-trifluoromethylphenyl)-1,4-dihydropyridine-3,5-carboxylate (**10**). Yield 22%, yellow-orange powder; mp 131–132 °C; ^1^H NMR (DMSO-d_6_) δ = 3.53 (s, 6H, COOCH_3_), 3.73, 3.74 (2 × s, 2 × 3H, OCH_3_), 4.70 (s, 2H, CH_2_), 4.79 (s, 1H, 4-H), 6.88 (dd, J = 8.2, 2.0 Hz, 1H, 6-H of benzyl), 6.97 (s, 1H, 2-H of benzyl), 6.99 (d, J = 8.2 Hz, 1H, 5-H of benzyl), 7.00 (d, J = 2.0 Hz, 1H, 2-H of benzyl), 7.31 (d, J = 8.2 Hz, 2H, 2-, 6-H of phenyl), 7.51 (s, 2H, 2-, 6-H), 7.54 (d, J = 8.1 Hz, 2H, 3-, 5-H); *m*/*z* (ESI) 514.49 (M + Na^+^).

Dimethyl 4-(3,4-difluorophenyl)-N-(3-methoxybenzyl)-1,4-dihydropyridine-3,5-carboxylate (**11**). Yield 22%, yellow powder; mp 99–100 °C; ^1^H NMR (DMSO-d_6_) δ = 3.54 (s, 6H, COOCH_3_), 3.74 (s, 3H, OCH_3_), 4.70 (s, 1H, 4-H), 4.76 (s, 2H, CH_2_), 6.86–6.93 (m, 3H, 2-, 4-, 6-H of benzyl), 6.93–7.01 (m, 2H, 2-, 5-H of phenyl), 7.18 7.27(m, 1H, 5-H of benzyl), 7.29–7.35 (m, 1H, 6-H of phenyl), 7.50 (s, 2H, 2-, 6-H); m/z (ESI) 452.63 (M + Na^+^).

Dimethyl 4-(3,4-difluorophenyl)-N-(4-methoxybenzyl)-1,4-dihydropyridine-3,5-carboxylate (**12**). Yield 46%, yellow crystals; mp 129–130 °C; mp 129–130 °C; ^1^H NMR (DMSO-d_6_) δ = 3.54 (s, 6H, COOCH_3_), 3.74 (s, 3H, OCH_3_), 4.68 (s, 1H, 4-H), 4.71 (s, 2H, CH_2_), 6.86–6.70 (m, 4H, 2-, 3-, 5, 6-H of benzyl), 7.17–7.33 (m, 3H, 2-, 5-, 6-H of phenyl), 7.49 (s, 2H, 2-, 6-H); *m*/*z* (ESI) 452.46 (M + Na^+^).

Dimethyl 4-(3,4-difluorophenyl)-N-(3,4-dimethoxybenzyl)-1,4-dihydropyridine-3,5-carboxylate (**13**). Yield 49%, white-yellow solid; mp 164–165 °C; ^1^H NMR (DMSO-d_6_) δ = 3.54 (s, 6H, COOCH_3_), 3.73, 3.74 (2 × s, 2 × 3H, OCH_3_), 4.69 (s, 2H, CH_2_), 4.70 (s, 1H, 4-H), 6.88 (dd, J = 8.2, 2.0 Hz, 1H, 6-H of benzyl), 6.92–7.01 (m, 4H, 2-, 5-H of benzyl and 2-, 5-H of phenyl), 7.24 (dd, J = 8.0, 2.1 Hz, 1H 6-H of phenyl), 7.49 (s, 2H, 2-, 6-H); *m*/*z* (ESI) 460.48 (M + H^+^).

Dimethyl N-(3,4-dimethoxybenzyl)-4-(3-fluorophenyl)-1,4-dihydropyridine-3,5-carboxylate (**14**). Yield 29%, yellow crystals; mp 135–136 °C; ^1^H NMR (DMSO-d_6_) δ = 3.54 (s, 6H, COOCH_3_), 3.73, 3.74 (2 × s, 2 × 3H, OCH_3_), 4.69 (s, 2H, CH_2_), 4.72 (s, 1H, 4-H), 6.80 (dd, J = 8.4, 2.1 Hz, 1H, 6-H of benzyl), 6.88 (dd, J = 8.2, 2.1 Hz, 1H, 6-H of phenyl), 6.88–7.00 (m, 4H, 2-, 5-H of benzyl and 2-, 4-H of phenyl), 7.16–7.25 (m, 1H, 5-H of phenyl), 7.49 (s, 2H, 2-, 6-H); *m*/*z* (ESI) 464.76 (M + H^+^).

Dimethyl N-(3,4-dimethoxybenzyl)-4-(4-fluorophenyl)-1,4-dihydropyridine-3,5-carboxylate (**15**). Yield 22%, yellow-orange powder; mp 159–161 °C; ^1^H NMR (DMSO-d_6_) δ = 3.53 (s, 6H, COOCH_3_), 3.73, 3.74 (2 × s, 2 × 3H, OCH_3_), 4.69 (s, 2H, CH_2_), 4.70 (s, 1H, 4-H), 6.87 (dd, J = 8.5, 2.0 Hz, 1H, 6-H of benzyl), 6.97 (s, 1H, 2-H of benzyl), 6.97 (d, J = 8.6 Hz, 1H, 5-H of benzyl), 6.99 (d, J = 8.6 Hz, 2H, 3-, 5-H of phenyl), 7.11 (d, J = 8.4 Hz, 1H, 2-H of phenyl), 7.12 (d, J = 8.5 Hz, 1H, 6-H of phenyl), 7.46 (s, 2H, 2-, 6-H); *m*/*z* (ESI) 464.76 (M + H^+^).

Dimethyl 4-(4-methoxy-3-trifluoromethylphenyl)-N-(3-trifluoromethylbenzyl)-1,4-dihydro-pyridine-3,5-carboxylate (**16**). Yield 49%, yellow solid; mp 155–156 °C; ^1^H NMR (DMSO-d_6_) δ = 3.54 (s, 6H, COOCH_3_), 3.81 (s, 3H, OCH_3_), 4.71 (s, 1H, 4-H), 4.90 (s, 2H, CH_2_), 7.06 (d J = 8.7 Hz, 1H, 5-H of phenyl), 7.18 (d, J = 2.3 Hz, 1H, 2-H of phenyl), 7.34 (dd, J = 8.7, 2.3 Hz, 1H, 6-H of phenyl), 7.58 (s, 2H, 2-, 6-H), 7.62-7.68 (m, 2H, 4-, 5-H of benzyl), 7.70–7.77 (m, 2H, 2-, 6-H of benzyl); m/z (ESI) 552.54 (M + Na^+^).

Dimethyl N-(3-methoxybenzyl)-4-(4-methoxy-3-trifluoromethylphenyl)-1,4-dihydropyridine-3,5-carboxylate (**17**). Yield 45%, yellow-white solid; mp 142–144 °C; ^1^H NMR (DMSO-d_6_) δ = 3.54 (s, 6H, COOCH_3_), 3.74, 3.82 (2 × s, 2 × 3H, OCH_3_), 4.71 (s, 1H, 4-H), 4.76 (s, 2H, CH_2_), 6.85-6.95 (m, 3H, 2-, 4-, 6-H of benzyl), 7.08 (d, J = 8.6 Hz, 1H, 5-H of phenyl), 7.26 (d, J = 2.3 Hz, 1H, 2-H of phenyl), 7.28–7.33 (m, 1H, 5H of benzyl), 7.35 (dd, J = 8.6, 2.3 Hz, 1H, 6-H of phenyl), 7.50 (s, 2H, 2-, 6-H); *m*/*z* (ESI) 514.79 (M + Na^+^).

Dimethyl N-(4-methoxybenzyl)-4-(4-methoxy-3-trifluoromethylphenyl)-1,4-dihydropyridine-3,5-carboxylate (**18**). Yield 41%, yellow-white crystals; mp 152–153 °C; ^1^H NMR (DMSO-d_6_) δ = 3.53 (s, 6H, COOCH_3_), 3.74, 3.81 (2 × s, 2 × 3H, OCH_3_), 4.68 (s, 1H, 4-H), 4.71 (s, 2H, CH_2_), 6.94 (dd, J = 6.5, 2.0 Hz, 2H, 3-, 5-H of benzyl), 7.07 (d, J = 8.7 Hz, 1H, 5-H of phenyl), 7.22 (d, J = 2.3 Hz, 1H, 2-H of phenyl), 7.28 (dd, J = 6.5, 2.0 Hz, 2H, 2-, 6-H of benzyl), 7.32 (dd, J = 8.7, 2.3 Hz, 1H, 6-H of phenyl), 7.49 (s, 2H, 2-, 6-H); *m*/*z* (ESI) 514.76 (M + Na^+^).

Dimethyl N-(3,4-dimethoxybenzyl)-4-(4-methoxy-3-trifluoromethylphenyl)-1,4-dihydro-pyridine-3,5-carboxylate (**19**). Yield 32%, yellow solid; mp 142–143 °C; ^1^H NMR (DMSO-d_6_) δ = 3.54 (s, 6H, COOCH_3_), 3.82, 3.83, 3.88 (3 × s, 3 × 3H, OCH_3_), 4.77 (s, 2H, CH_2_), 4.86 (s, 1H, 4-H), 6.96–7.01 (m, 2H, 5-, 6-H of benzyl), 7.04 (d, J = 8.5 Hz, 1H, 5-H of phenyl), 7.07 (d, J = 1.8 Hz, 1H, 2-H of benzyl), 7.18 (d, J = 2.3 Hz, 1H, 2-H of phenyl), 7.43 (dd, J = 8.5, 2.3 Hz, 1H, 6-H of phenyl), 7.47 (s, 2H, 2-, 6-H); *m*/*z* (ESI) 544.54 (M + Na^+^).

Dimethyl 4-(3,4-dimethoxyphenyll)-N-(3-trifluoromethylbenzyl)-1,4-dihydropyridine-3,5-carboxylate (**20**). Yield 55%, white-yellow solid; mp 161–163 °C; ^1^H NMR (DMSO-d_6_) δ = 3.50 (s, 6H, COOCH_3_), 3.53, 3.66 (2 × s, 2 × 3H, OCH_3_), 4.64 (s, 1H, 4-H), 4.89 (s, 2H, CH_2_), 6.58 (dd, J = 8.2, 2.0 Hz, 1H, 6-H of phenyl), 6.60 (d, J = 2.0 Hz, 1H, 2-H of phenyl), 6.71 (d, J = 8.2 Hz, 1H, 5-H of phenyl), 7.54 (s, 2H, 2-, 6-H), 7.62–7.73 (m, 3H, 3-, 5-, 6-H of benzyl), 7.77 (t, J = 1.6 Hz, 1H, 2-H of benzyl); *m*/*z* (ESI) 514.12 (M + Na^+^).

Dimethyl 4-(3,4-dibenzyloxyphenyll)-N-(3-trifluoromethylbenzyl)-1,4-dihydropyridine-3,5-carboxylate (**21**). Yield 6%, yellow solid; mp 97–98 °C; ^1^H NMR (DMSO-d_6_) δ = 3.57 (s, 6H, COOCH_3_), 4.81 (s, 1H, 4-H), 4.99 (s, 2H, NCH_2_), 5.02, 5.08 (2 × s, 2 × 2H; OCH_2_), 6.76 (dd, J = 8.3, 2.1 Hz, 1H, 6-H of phenyl), 6.88 (d, J = 8.3 Hz, 1H, 5-H of phenyl), 6.99 (d, J = 2.1 Hz, 1H, 2-H of phenyl), 7.25–7.41 (m, 6H, 3-, 4-, 5-H of 4-OBz, 4-, 5-H of 3-OBz, 6-H of benzyl), 7.44–7.49 (m, 4H, 2-, 6-H of 4-OBz, 3-, 6-H of 3-OBz), 7.42 (s, 2H, 2-, 6-H), 7.62–77.78 (m, 4H, 2-H of 3-OBz, 2-, 4-, 5-H of benzyl); *m*/*z* (ESI) 666.71 (M + Na^+^).

### 3.3. MRP Inhibition Assay

The human pancreatic carcinoma cell lines colo357 and colo357 MRP4 that resulted from transfection of the colo357 cells with an MRP4 vector construct were used [49], and the MRP4 overexpression was confirmed by Western blot as shown in the Supplementary Materials. Both cell lines and the ovarian carcinoma cell line A2780 were cultured in RPMI-1640 medium that was supplemented with fetal calf serum (10%), minimal essential amino acids (1%), and penicillin/streptomycin (1%) at 37 °C and under carbon dioxide atmosphere (5%).

In the assay, every 200.000 cells were given in an Eppendorf tube. The cells were centrifuged at 2000 RPM at 4 °C. The supernatant was removed, and the samples were stored on ice. Then they were resuspended in RPMI-1640 medium, and test compounds and MK571 or probenecid control were added from stock solutions of 4000 µM in DMSO to give a final concentration of 10 µM. DMSO was used as control with no effect on the fluorescence. The samples were cultured at 37 °C for 20 min and 1200 RPM in a thermomixer. Then the fluorescent calcein was added from a PBS solution to give a final concentration of 0.005 µM in the colo cells, and the fluorescent CFDA was added from a PBS solution with the same final concentration to the ovarian cells. The samples were centrifuged again, and the supernatant was removed. Then PBS was added, and the samples were centrifuged again. The washing procedure was repeated. Finally, the fluorescence of the resuspended cells was measured by flow cytometry using 10.000 cells and a MACSQuant Analyzer (Bergisch Gladbach, Germany). The measurement was conducted three times each for inhibitor-treated and untreated cells of both cell lines. The MRP4 *FAR* values were calculated as the ratio of the fluorescence of the treated colo357 MRP4 to the treated colo357 cells with each value corrected by the fluorescence of the untreated colo cells. The MRP1 *FAR* values were calculated as the ratio of the fluorescence of the treated ovarian cells related to the untreated control cells.

### 3.4. MRP4 Reversal Assay

10.000 cells of each cell line were cultured in wells of a 96-well plate at 37 °C under a carbon dioxide atmosphere (5%) for 24 h. Increasing concentrations of the MRP4 substrate 6-mercaptopurine from 0.0001 µM to finally 100 µM were added after the test compound and MK571, each at a concentration of 10 µM, had been added. The plate was incubated for 48 h under the original culture conditions. Then the MTT reagent was added to each well (10 µL of a stock solution of 5 mg/mL in PBS) and incubation continued for 4 h. Then 100 µL DMSO was added to each well to solve the formazan reduction product. The plate was shaken for 30 min on a plate shaker and, finally, the formazan absorption was measured. The described method was repeated three times. The IC_50_ values were determined from the resulting sigmoid curves.

## 4. Conclusions

MDR is the main problem in anticancer treatment, especially in the case of metastatic cancer that generally cannot be treated as it would afford the effectiveness of various anticancer drugs. As novel anticancer drugs are specific to single cancer drug targets, their use is limited to certain kinds of cancer but not for the treatment of MDR. Moreover, these novel drugs also become affected by the MDR phenomenon. The aim to block the MDR by the use of effective drugs is still a challenge. If a respective tumor expresses a transmembrane efflux pump as the main cause of the MDR phenomenon, the use of an inhibitor will treat the MDR and resensitize the tumor toward the anticancer drug treatment.

We developed a novel class of MRP4 inhibitors that was evaluated to block the MRP4 activity toward the fluorescent MRP4 substrate calcein. Certain substituent-dependent effects were observed with best activities for a disubstituted 4-phenyl residue and a *meta* substituted *N*-benzyl ring residue at the symmetric molecular scaffold. All compounds showed a mainly better activity than the used MRP4 inhibitor MK571. In order to further profile our compound class, the best inhibitor was investigated to restore the MRP4-mediated anticancer drug toxicity in the used MRP4-overexpressing cell line model. The use of that compound almost reached the anticancer drug toxicity determined in the non-MRP4-expressing cell line. Thus our novel compound class is promising for further studies to perspectively combat MRP4-mediated anticancer drug resistance.

## Figures and Tables

**Table 1:** MRP4 inhibition data of target compounds **4**–**21** with varied substitution patterns expressed as *FAR* values.

Compound	R^1^	R^2^	R^3^	R^4^	*FAR*
Value ^[a]^					
**4**	CF_3_	H	CF_3_	H	1.28
**5**	CF_3_	H	OMe	H	1.21
**6**	CF_3_	H	H	OMe	1.11
**7**	CF_3_	H	OMe	OMe	1.10
**8**	H	CF_3_	OMe	H	1.28
**9**	H	CF_3_	H	OMe	1.11
**10**	H	CF_3_	OMe	OMe	1.26
**11**	F	F	OMe	H	1.43
**12**	F	F	H	OMe	1.40
**13**	F	F	OMe	OMe	1.23
**14**	F	H	OMe	OMe	1.21
**15**	H	F	OMe	OMe	1.12
**16**	CF_3_	OMe	CF_3_	H	1.19
**17**	CF_3_	OMe	OMe	H	1.55
**18**	CF_3_	OMe	H	OMe	0.95
**19**	CF_3_	OMe	OMe	OMe	1.22
**20**	OMe	OMe	CF_3_	H	0.98
**21**	OBn	OBn	CF_3_	H	1.30

^[a]^ Fluorescence activity ratio as mean of three determinations.

## Supplementary Materials

The following are available online, Western blot of MRP4 overexpression.

## Sample Availability

Samples of the compounds are not available from the authors.
