# Correction: Döring et al. Discovery of Novel Symmetrical 1,4-Dihydropyridines as Inhibitors of Multidrug-Resistant Protein (MRP4) Efflux Pump for Anticancer Therapy. *Molecules* 2021, *26*, 18

**DOI:** 10.3390/molecules27113575

**Published:** 2022-06-02

**Authors:** Henry Döring, David Kreutzer, Christoph Ritter, Andreas Hilgeroth

**Affiliations:** 1 Research Group of Drug Development, Institute of Pharmacy, Martin Luther University Halle-Wittenberg, 06120 Halle, Germany; henry.doering@arcor.de (H.D.); david.kreutzer94@web.de (D.K.); 2 Department of Clinical Pharmacy, Institute of Pharmacy, Ernst Moritz Arndt University Greifswald, 17489 Greifswald, Germany; ritter@uni-greifswald.de

The authors would like to correct an error made through no fault of their own in the title paper [1]. The error is related to the *FAR* values in Table 1. In the earlier published version, the *FAR* values of compounds **15**–**21** were wrongly printed. So, they had to be corrected, from 1.19 to 1.12 for **15**, 1.55 to 1.19 for **16**, 0.95 to 1.55 for **17**, 1.22 to 0.95 for **18**, 0.98 to 1.22 for **19**, 1.30 to 0.98 for **20** and 0.82 to 1.30 for **21**. We provide below the corrected Table 1. The change has no influence on the reported results. The original article has been updated. The authors would like to apologize for any inconvenience caused to the readers by this change.

## Figures and Tables

**Table 1:** MRP4 inhibition data of target compounds **4**–**21** with varied substitution patterns expressed as *FAR* values.

Compound	R^1^	R^2^	R^3^	R^4^	*FAR*
Value ^[a]^					
**4**	CF_3_	H	CF_3_	H	1.28
**5**	CF_3_	H	OMe	H	1.21
**6**	CF_3_	H	H	OMe	1.11
**7**	CF_3_	H	OMe	OMe	1.10
**8**	H	CF_3_	OMe	H	1.28
**9**	H	CF_3_	H	OMe	1.11
**10**	H	CF_3_	OMe	OMe	1.26
**11**	F	F	OMe	H	1.43
**12**	F	F	H	OMe	1.40
**13**	F	F	OMe	OMe	1.23
**14**	F	H	OMe	OMe	1.21
**15**	H	F	OMe	OMe	1.12
**16**	CF_3_	OMe	CF_3_	H	1.19
**17**	CF_3_	OMe	OMe	H	1.55
**18**	CF_3_	OMe	H	OMe	0.95
**19**	CF_3_	OMe	OMe	OMe	1.22
**20**	OMe	OMe	CF_3_	H	0.98
**21**	OBn	OBn	CF_3_	H	1.30

^[a]^ Fluorescence activity ratio as mean of three determinations.
