# Supplementary material for: Discovery of Novel Symmetrical 1,4-Dihydropyridines as Inhibitors of Multidrug-Resistant Protein (MRP4) Efflux Pump for Anticancer Therapy
Source: Molecules. 2020 Dec 22;26(1):18. doi: 10.3390/molecules26010018 (PMC7793087; doi:10.3390/molecules26010018)
Supplement: Supplementary file 1 [file molecules-26-00018-s001.pdf]

Supplementary Materials

Communication

Discovery of Novel Symmetrical 1,4-Dihydropyridines as Inhibitors of Multidrug-Resistant Protein (MRP4) Efflux Pump for Anticancer Therapy

Henry Döring [1], David Kreutzer [1], Christoph Ritter [2] and Andreas Hilgeroth [1,*]

[1] Institute of Pharmacy, Research Group of Drug Development, Martin Luther University Halle-Wittenberg; andreas.hilgeroth@pharmazie.uni-halle.de

[2] Institute of Pharmacy, Department of Clinical Pharmacy, Ernst Moritz Arndt University Greifswald; ritter@uni-greifswald.de

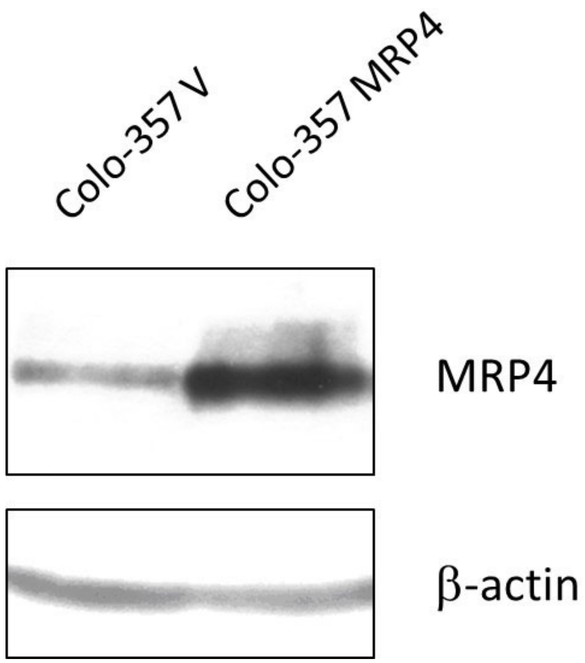

Colo-357 V
Colo-357 MRP4
MRP4
β-actin
